# The Efficacy of Linked Color Imaging in the Endoscopic Diagnosis of Barrett's Esophagus and Esophageal Adenocarcinoma

**DOI:** 10.1155/2020/9604345

**Published:** 2020-09-29

**Authors:** Mamoru Tokunaga, Tomoaki Matsumura, Kentaro Ishikawa, Tatsuya Kaneko, Hirotaka Oura, Tsubasa Ishikawa, Ariki Nagashima, Wataru Shiratori, Kenichiro Okimoto, Naoki Akizue, Daisuke Maruoka, Yuki Ohta, Keiko Saito, Tomoo Nakagawa, Tetsuhiro Chiba, Makoto Arai, Jun Kato, Naoya Kato

**Affiliations:** ^1^Department of Gastroenterology, Graduate School of Medicine, Chiba University Chiba, Japan; ^2^Department of Medical Oncology, Chiba University Chiba, Japan

## Abstract

**Background:**

The present study aimed to evaluate the efficacy of linked color imaging (LCI) in diagnosing Barrett's esophagus (BE) and esophageal adenocarcinoma (EAC).

**Methods:**

A total of 112 and 12 consecutive patients with BE and EAC were analyzed. The visibility scores of BE and EAC ranging from 4 (excellent visibility) to 0 (not detectable) were evaluated by three trainees and three experts using white light imaging (WLI), LCI mode, and blue laser imaging bright (BLI-b) mode. In addition, L∗a∗b∗ color values and color differences (ΔE∗) were evaluated using the CIELAB color space system.

**Results:**

The visibility score of the BE in LCI mode (2.94 ± 1.32) was significantly higher than those in WLI (2.46 ± 1.48) and BLI-b mode (2.35 ± 1.46) (*p* < 0.01). The color difference (ΔE∗) from the adjacent gastric mucosa in LCI mode (17.11 ± 8.53) was significantly higher than those in other modes (12.52 ± 9.37 in WLI and 11.96 ± 6.59 in BLI-b mode, *p* < 0.01). The visibility scores of EAC in LCI mode (2.56 ± 1.47) and BLI-b mode (2.51 ± 1.28) were significantly higher than that in WLI (1.64 ± 1.46) (*p* < 0.01). The color difference (ΔE∗) from the adjacent normal Barrett's mucosa in LCI mode (19.96 ± 7.97) was significantly higher than that in WLI (12.95 ± 11.86) (*p* = 0.03).

**Conclusion:**

The present findings suggest that LCI increases the visibility of BE and EAC and contributes to the improvement of the detection of these lesions.

## 1. Introduction

Barrett's esophagus (BE) is pathologically defined as a columnar epithelium that replaces the squamous epithelium of the esophagogastric junction (EGJ) during the process of healing from esophagitis [1, 2], and it is a precursor of esophageal adenocarcinoma (EAC) [3]. In western countries, EAC, including Barrett's EAC, accounts for approximately 60% of all esophageal cancers, and it is considered an important disease due to its poor prognosis [4, 5]. When Barrett's EAC is detected based on symptoms, the 5-year survival of patients is less than 10% [6]. In Japan, the incidence of EAC has recently increased [7, 8]. In recent reports from Japan, the annual carcinogenic risk of EAC in patients with BE ≥3 cm was approximately 1.2% annually [9], and this percentage was higher than that of western countries at 0.31% (0.21 % -0.40 %) [10]. The prognosis of EAC is poor when it is diagnosed during the advanced stage. However, if it can be detected at an early stage, endoscopic resection can be conducted. Recently, the efficacy of endoscopic resection, such as endoscopic submucosal dissection (ESD) or endoscopic mucosal resection (EMR), has been reported worldwide, and a favorable long-term prognosis after treatment has been obtained [11–13]. To detect EAC at an early stage, an appropriate endoscopic surveillance is required. However, early-stage EAC arising from an inflamed BE is still challenging to diagnose [14, 15]. Therefore, a random four-quadrant biopsy is recommended in western countries [16].

In recent years, the linked color imaging (LCI) system has been developed as a new endoscopic imaging modality [17]. LCI uses band laser (wavelength 410 ± 10 nm) in addition to white-light laser; therefore, the LCI mode helps emphasize vascular and surface structures and color differences while maintaining a bright vision [17]. The efficacy of LCI in the diagnosis of active *Helicobacter pylori* infection [18], atrophic gastritis [19], gastric cancer [20–22], colon cancer [23], and short-segment BE (SSBE) [24] in clinical practice has been reported. However, whether LCI is effective in diagnosing long segment BE (LSBE), and EAC has not been elucidated. The present study aimed to validate the efficacy of LCI in the diagnosis of BE including LSBE and early-stage EAC.

## 2. Materials and Methods

### 2.1. Patients

This study used endoscopic images of consecutive BE and early-stage EAC at our institution between April 2017 and March 2019. Patients with advanced EACs were excluded from this study as it can be easily recognized via endoscopy. Finally, 112 and 12 consecutive patients with BE and early-stage EAC were evaluated. BE was defined as a columnar-lined epithelium (CLE) diagnosed via endoscopy. CLE was identified in the region from the EGJ to the squamocolumnar junction. EGJ was defined as the distal end of the lower esophageal palisade vessels [25]. If the vessels were not visible, EGJ was defined as the proximal end of the gastric folds [25]. The presence of intestinal metaplasia was not used to diagnose BE according to the Japanese definition [25]. This study was reviewed and approved by the institutional review board of Chiba University School of Medicine and was conducted in accordance with the principles of the Declaration of Helsinki. In addition, this study was registered at the University Hospital Medical Information Network (UMIN000027187).

### 2.2. Esophagogastroduodenoscopy

Esophagogastroduodenoscopy was conducted using the LASEREO system with an EG-L600WR7 or EG-L600ZW7 endoscope (FUJIFILM, Tokyo, Japan). Endoscopic images were taken at the same site in three modes: white light imaging (WLI), LCI mode, and blue laser imaging bright (BLI-b) mode. LSBE and SSBE were defined as ≥3 cm and <3 cm in length of BE, respectively. EACs were resected via endoscopically (ESD or EMR) and were pathologically confirmed as Barrett's EAC.

### 2.3. Visibility Score of BE

To investigate the efficacy of LCI mode in the diagnosis of BE, we selected one image acquired using WLI, LCI mode, and BLI-b mode at the same site in a fully extended condition without zooming. A total of 336 images were prepared for each modality and were presented consecutively to six endoscopists for interpretation. The six endoscopists included three experts and three trainees. In this study, we defined an expert as an endoscopist with ≥5 years of experience in using image-enhanced endoscopy (IEE) and a trainee as an endoscopist with <1 year of experience. The images were shown to each individual endoscopist in a random order on a black background with a same size on a power point. The evaluation of the visibility score of BE and EAC was performed by using a previously reported visibility score as follows: 4, excellent visibility (easily detectable); 3, good visibility (detectable with cautious observation); 2, fair visibility (hardly detectable without cautious examination); and 1, poor visibility (not detectable without repeated cautious examination) [23, 26]. In this study, in addition to the abovementioned four points rating, we added the score of 0, which indicated that the lesions were not detectable.

### 2.4. L∗a∗b∗ Color Values of BE and Color Differences (ΔE∗) of BE from Adjacent Gastric Mucosa

To evaluate the color differences between BE and adjacent gastric mucosa, the images were assessed and scored for an objective evaluation based on *L*∗*a*∗*b*∗ (*L*∗ = light/dark; *a*∗ = red/green; and *b*∗ = yellow/blue) color values in the CIELAB color space system [27] using the Adobe® Photoshop CC 2017, as previously described [24, 28]. The color difference (Δ*E*∗ = [(Δ*L*∗)^2^ + (Δ*a*∗)^2^ + (Δ*b*∗)^2^]^1/2^) of the pixel values based on *L*∗*a*∗*b*∗ color spaces within the region of interest was analyzed to evaluate the visibility of each color image [24, 28].

### 2.5. Visibility Score of EAC

In the same way as in the BE case, in total, 36 images were prepared for each mode and were assessed by six endoscopists (three experts and three trainees) using the visibility score (0–4).

### 2.6. L∗a∗b∗ Color Values of EAC and Color Differences (ΔE∗) of EAC from Adjacent Normal Barrett's Mucosa

For the analysis of EAC, color differences (ΔE∗) between EAC and the adjacent normal Barrett's mucosa, but not the difference between EAC and the adjacent gastric mucosa, were evaluated.

### 2.7. Sample Size

Sample size was calculated using the PASS software 2020 (NCSS Inc., Kaysville, UT, the USA). To ensure that the sample size for the patients with LSBE and those with EAC were adequate for estimations, preliminary data (5 patients each in the beginning) were used. Regarding the sample size for the patients with LSBE, the mean of paired color differences (ΔE∗) in WLI and LCI mode of 6.3 with an estimated standard deviation of paired differences of 5.9. If the true difference in the WLI and LCI mode means is equal to 6.3, the study would require a sample of 10 patients with LSBE to be able to reject the null hypothesis (the population means of the WLI and LCI mode groups were equal) using a two-sided paired Wilcoxon signed-rank test with a probability of 80% and at a significance level of 0.05. On the other hand, regarding the sample size for the patients with EAC, the mean of paired color differences (ΔE∗) in WLI and LCI mode of 8.1 with an estimated standard deviation of paired differences of 8.5. If the true difference in the WLI and LCI mode means is equal to 8.1, the study would require a sample of 12 patients with EAC in order to be able to reject the null hypothesis using a two-sided paired Wilcoxon signed-rank test with a probability of 80% and at a significance level of 0.05.

### 2.8. Statistical Analysis

Baseline data were presented as mean ± standard deviation (SD). The sample size was small, especially for EACs and LSBEs. Furthermore, the data of total BEs were not normally distributed. Based on these facts, the differences in visibility scores and color differences (ΔE∗) among the groups were analyzed using a Wilcoxon signed-rank test. All statistical analyses were performed using the Statistical Package for the Social Sciences software version 26 (SPSS Inc., Chicago, IL, the USA). *p* values <0.05 were considered statistically significant.

## 3. Results

### 3.1. Patients

**Figure 1:**
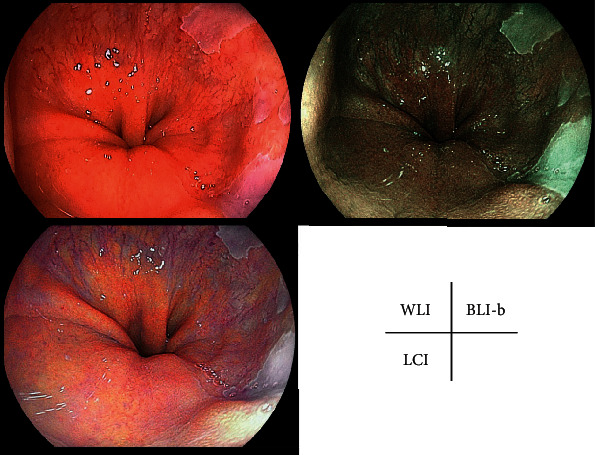
Representative case of short-segment Barrett's esophagus. WLI, LCI mode, and BLI-b mode at the same site in a fully extended condition without zooming. WLI: white light imaging; LCI: linked color imaging; BLI-b: blue laser imaging bright.

**Figure 2:**
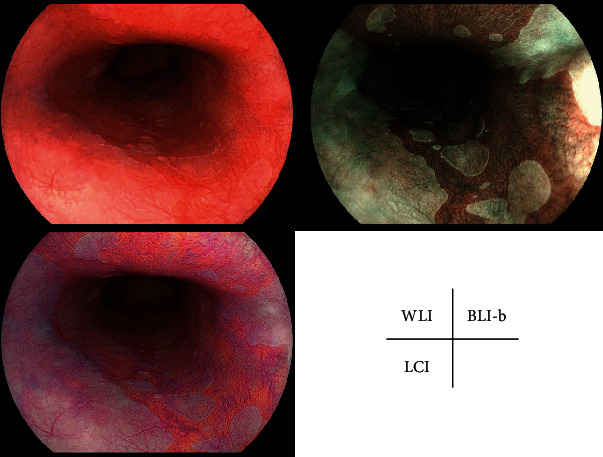
Representative case of long-segment Barrett's esophagus. WLI: white light imaging; LCI: linked color imaging; BLI-b: blue laser imaging bright.

**Figure 3:**
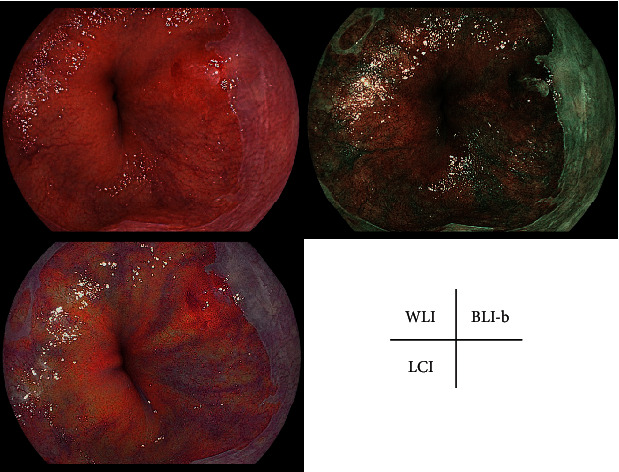
Representative case of esophageal adenocarcinoma. WLI, LCI mode, and BLI-b mode at the same site in a fully extended condition without zooming. Esophageal adenocarcinoma was resected via ESD and was pathologically confirmed as Barrett's adenocarcinoma.

The characteristics of the patients with BE and early-stage EAC are shown in Table 1. Representative cases with SSBE, LSBE, and early-stage EAC are shown in Figures 1–3.

### 3.2. Visibility Score and Color Difference of BE from Adjacent Gastric Mucosa

The mean visibility scores (± SD) of BE in WLI, LCI, and BLI-b modes are shown in Table 2. The scores obtained in LCI mode were significantly higher than those in WLI (*p* < 0.01) and BLI-b mode (*p* < 0.01). This difference was recognized by both trainees (*p* < 0.01) and experts (*p* < 0.01). When SSBE and LSBE were examined separately, this difference was also recognized in both SSBE and in LSBE (*p* < 0.01).

**Figure 4:**
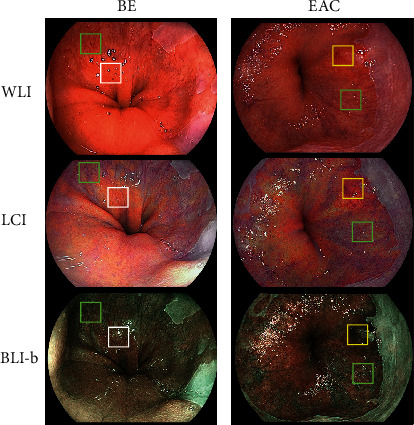
Representative cases for evaluating color difference. The color difference (ΔE∗) of the pixel values based on *L*∗*a*∗*b*∗ color spaces within the region of interest was analyzed to evaluate the visibility of each color image. Green line squares indicate region of interest in normal Barrett's mucosa. White line squares indicate region of interest in gastric mucosa. Yellow line squares indicate region of interest in normal Barrett's mucosa. BE: Barrett's esophagus; EAC: esophageal adenocarcinoma; WLI: white light imaging; LCI: linked color imaging; BLI-b: blue laser imaging bright.

Representative cases for analyzing color difference are shown in Figure 4. The color differences (ΔE∗) of BE from the adjacent gastric mucosa in WLI, LCI, and BLI-b modes are shown in Table 3. Color differences (ΔE∗) from the adjacent gastric mucosa were 12.52 ± 9.37 in WLI, 17.11 ± 8.53 in LCI mode, and 11.96 ± 6.59 in BLI-b mode. The difference (ΔE∗) in LCI mode was significantly higher than those in WLI (*p* < 0.01) and BLI-b mode (*p* < 0.01). When SSBE and LSBE were examined separately, this difference was noted in both SSBE (13.17 ± 9.60 in WLI, 17.29 ± 8.83 in LCI mode, and 12.12 ± 6.75 in BLI-b mode, *p* < 0.01 and *p* < 0.01, respectively) and in LSBE (6.48 ± 2.98 in WLI, 15.43 ± 4.85 in LCI mode, and 10.47 ± 4.93 in BLI-b mode, *p* = 0.013 and *p* = 0.028, respectively).

### 3.3. Visibility Score and Color Difference of EAC from Adjacent Normal Barrett's Mucosa

The mean visibility scores (± SD) of EAC in WLI, LCI, and BLI-b modes are shown in Table 4. The visibility score of EAC in LCI mode was significantly higher than that in WLI. This difference was recognized in both the trainees (*p* < 0.01) and experts (*p* < 0.01). The visibility score of EAC in BLI-b mode was also significantly higher than that in WLI. No statistical differences were observed between that in LCI mode and that in BLI-b mode (*p* = 0.773).

The color differences (ΔE∗) of EAC from the adjacent normal Barrett's mucosa in WLI, LCI, and BLI-b modes are shown in Table 5. In LCI mode, the difference (ΔE∗) was significantly higher than that in WLI (*p* = 0.034). When EAC in SSBE and that in LSBE were examined separately, the significant color difference in LCI mode to WLI was recognized only in SSBE (*p* = 0.012).

## 4. Discussion

In the present study, we showed that the use of LCI improves the visibility of both BE and EAC. This report first assessed the beneficial effects of LCI in patients with BE including LSBE, and those with EAC.

To accurately evaluate the efficacy of LCI, we evaluated not only visibility but also color difference using the CIELAB color space system for an objective evaluation. In addition, the evaluations were carried out not only by experts who are familiar with the use of IEE but also by trainees with little experience in the use of IEE. The visibility scores for BE and EAC in LCI mode were significantly higher than those in WLI in trainees as well as experts, and therefore, it may be useful for the diagnosis of BE and EAC in screening tests performed by trainees unfamiliar with IEE.

Regarding the efficacy of LCI on BE, Takeda et al. have reported that the use of LCI improves the visibility of SSBE [24], and our study further supported the results of the previous study. In their study, they evaluated 63 patients with SSBE and color difference using the CIELAB color space system, while we evaluated 112 patients with BE, including 10 patients with LSBE. Since we included a larger number of consecutive patients with BE, including LSBE, we believe that we were able to show more reliable data. Furthermore, we also examined not only patients with BE but also consecutive 12 patients with EAC.

EAC in BE is often challenging to detect due to the characteristics of the BE mucosa that arise from inflammation [14, 15]. In addition, most early-stage EACs were reported as flat lesions, not protruding lesions [7, 11]. For these reasons, EAC is known to be one of the most difficult cancers to detect endoscopically. Gastric cancer is also one of the difficult cancers to detect endoscopically, because it also arises from inflamed mucosa. The usefulness of LCI for gastric cancer visibility has been studied and reported [21]. Until recently, there have been no prospective studies on the detection of gastric cancer. However, very recently, Gao et al. conducted a randomized trial comparing gastric cancer detection rates in the LCI+WLI and WLI groups and reported that the detection rate of gastric cancers was higher in the LCI+WLI group than in the WLI group [29]. Our results on the usefulness of LCI for EAC suggest that LCI may be useful in the detection of EAC as well, and similar prospective randomized controlled trials are desirable in the future. In our study, when EAC in SSBE and that in LSBE were examined separately, despite visibility scores of EAC in LCI mode were significantly higher than that in WLI in both SSBE and LSBE, a statistically significant color difference was not observed in patients with LSBE. EACs in LSBE were reported to be less frequently reddish compared to EACs in SSBE [30], which may have affected these results. Recently, Nakamura et al. reported that LCI improved the visibility of esophageal squamous cell carcinoma [31]. Furthermore, they showed that its usefulness was higher in cases without background coloration. Since LCI can enhance slight differences in mucosal color, it is possible that the slight color differences in EAC present in LSBE may have contributed to the improved visibility. Since there were few patients with EAC in LSBE in this study, the usefulness of LCI for EAC in LSBE needs further investigation.

As mentioned above, EAC is difficult to detect endoscopically; therefore, the Seattle biopsy protocol has been used for endoscopic surveillance in patients with BE. However, the procedure involves 4-quadrant biopsy sampling of every 1 to 2 cm of the columnar-lined esophagus, which is time-consuming, labor-intensive, and costly. In addition, the burden on the pathologist is significant. In recent years, the efficacy of the target biopsies using acetic acid [32, 33] or Narrow Band Imaging (NBI) [34, 35] has been reported. In addition, several classifications of EAC using magnified NBI have been reported [36–38]. These classifications are very useful in clinical practice. On the other hand, these observations require some experience and time. If the use of nonmagnified LCI observation improves the diagnostic yield of EAC, this may change the manner of BE assessment in the future. Further prospective study about whether the use of LCI improves the diagnostic yield of EAC must be conducted.

Some genetic abnormalities in BE and EAC have also been recently reported [39, 40]. These genetic findings might lead to the stratification of risk for EAC and early detection of EAC. In this study, we only assessed the visibility of EAC using endoscopic images, but future endoscopic imaging evaluations that take into account the genetic background might lead to better risk assessment, early detection of lesions, and suggestions for appropriate follow-up methods.

The present study had several limitations. First, we used relatively little data obtained from retrospective cohorts in a single facility. This may have resulted in methodological biases. Although we calculated the sample sizes, the numbers of patients with LSBE and those with EAC were relatively small; thus, the outcome may have been influenced by one gross form of LSBE and EAC. Second, we did not confirm the presence of intestinal metaplasia, because it is not required for the diagnosis of BE in Japan [25]. Therefore, there is a possibility that the BE we defined in this study may not be diagnosed as BE in some countries [41]. Third, this study conducted an evaluation using still images. The actual diagnosis and detection of BE and EAC are conducted through video; thus, an accurate evaluation may be better with the use of a video.

## 5. Conclusions

The LCI has potential benefits, and it is a promising clinical diagnostic modality for EAC in patients with BE. This simple, affordable diagnostic tool may be useful in diagnosing patients with BE and EAC.

## Figures and Tables

**Table 1:** Characteristics of the patients.

	Patients with BE *n* = 112	Patients with EAC *n* = 12
Age (years, ± SD)	68.0 ± 15.2	70.9 ± 9.3
Male/female	81/31	9/3
SSBE/LSBE	102/10	8/4
Hiatal hernia (%)	57 (50.8%)	3 (25.0%)
Characteristics of the lesions in EAC		
Diameter, mean ± SD (mm)	—	21.4 ± 13.1
Paris type (IIa/IIc/IIb)	—	3/4/5
Invasion depth (m1-m2/m3-sm)	—	11/1

Data are presented as mean ± standard deviation (SD). BE: Barrett's esophagus; EAC: esophageal adenocarcinoma; SSBE: short-segment Barrett's esophagus; LSBE: long-segment Barrett's esophagus.

**Table 2:** The mean visibility scores of BE in WLI, LCI, and BLI-b modes.

	WLI	LCI	BLI-b	*P* value (WLI vs. LCI)	*P* value (WLI vs. BLI-b)	*P* value (LCI vs. BLI-b)
All BEs (*n* = 112)						
All, mean ± SD	2.46 ± 1.48	2.94 ± 1.32	2.35 ± 1.46	<0.01^∗^	0.019^∗^	<0.01^∗^
Expert, mean ± SD	2.22 ± 1.54	2.87 ± 1.40	2.17 ± 1.51	<0.01^∗^	0.49	<0.01^∗^
Trainee, mean ± SD	2.70 ± 1.39	3.02 ± 1.25	2.53 ± 1.38	<0.01^∗^	0.014^∗^	<0.01^∗^
SSBEs (*n* = 102)						
All, mean ± SD	2.44 ± 1.47	2.93 ± 1.31	2.32 ± 1.46	<0.01^∗^	0.023^∗^	<0.01^∗^
Expert, mean ± SD	2.15 ± 1.42	2.84 ± 1.38	2.09 ± 1.50	<0.01^∗^	0.482	<0.01^∗^
Trainee, mean ± SD	2.72 ± 1.37	3.02 ± 1.23	2.55 ± 1.37	<0.01^∗^	0.018^∗^	<0.01^∗^
LSBEs (*n* = 10)						
All, mean ± SD	2.62 ± 1.55	3.02 ± 1.43	2.54 ± 1.46	0.01^∗^	0.575	0.01^∗^
Expert, mean ± SD	2.78 ± 1.63	3.12 ± 1.53	2.78 ± 1.54	<0.01^∗^	1.00	0.013^∗^
Trainee, mean ± SD	2.45 ± 1.48	2.90 ± 1.33	2.30 ± 1.44	0.021^∗^	0.528	0.016^∗^

Wilcoxon signed-rank test, ^∗^*P* < 0.05. WLI: white light imaging; LCI: linked color imaging; BLI-b: blue laser imaging bright; BE: Barrett's esophagus; SSBE: short-segment Barrett's esophagus; LSBE: long-segment Barrett's esophagus; SD: standard deviation.

**Table 3:** The color differences (ΔE∗) of BE from the adjacent gastric mucosa in WLI, LCI, and BLI-b modes.

	WLI	LCI	BLI-b	*P* value (WLI vs. LCI)	*P* value (WLI vs. BLI-b)	*P* value (LCI vs. BLI-b)
All BEs, mean ± SD	12.52 ± 9.37	17.11 ± 8.53	11.96 ± 6.59	<0.01^∗^	0.31	<0.01^∗^
SSBEs, mean ± SD	13.17 ± 9.60	17.29 ± 8.83	12.12 ± 6.75	<0.01^∗^	0.49	<0.01^∗^
LSBEs, mean ± SD	6.48 ± 2.98	15.43 ± 4.85	10.47 ± 4.93	0.013^∗^	0.20	0.028^∗^

Wilcoxon signed-rank test, ^∗^*P* < 0.05. WLI: white light imaging; LCI: linked color imaging; BLI-b, blue laser imaging bright; BE: Barrett's esophagus; SSBE: short-segment Barrett's esophagus; LSBE: long-segment Barrett's esophagus; SD: standard deviation.

**Table 4:** The mean visibility scores of EAC in WLI, LCI, and BLI-b modes.

	WLI	LCI	BLI-b	*P* value (WLI vs. LCI)	*P* value (WLI vs. BLI-b)	*P* value (LCI vs. BLI-b)
All EACs, mean ± SD	1.64 ± 1.46	2.56 ± 1.47	2.51 ± 1.28	<0.01^∗^	<0.01^∗^	0.773
Expert, mean ± SD	1.33 ± 1.24	2.56 ± 1.44	2.28 ± 1.13	<0.01^∗^	<0.01^∗^	0.083
Trainee, mean ± SD	1.94 ± 1.62	2.56 ± 1.52	2.75 ± 1.40	<0.01^∗^	<0.01^∗^	0.111
EACs in SSBE, mean ± SD	2.29 ± 1.33	3.19 ± 1.04	2.94 ± 0.97	<0.01^∗^	<0.01^∗^	0.09
EACs in LSBE, mean ± SD	0.33 ± 0.56	1.29 ± 1.39	1.67 ± 1.43	<0.01^∗^	<0.01^∗^	0.013^∗^

Wilcoxon signed-rank test, ^∗^*P* < 0.05. WLI: white light imaging; LCI: linked color imaging; BLI-b: blue laser imaging bright; EAC: esophageal adenocarcinoma; SSBE: short-segment Barrett's esophagus; LSBE: long-segment Barrett's esophagus; SD: standard deviation.

**Table 5:** The color differences (ΔE∗) of EAC from the adjacent normal Barrett's mucosa in WLI, LCI, and BLI-b modes.

	WLI	LCI	BLI-b	*P* value (WLI vs. LCI)	*P* value (WLI vs. BLI-b)	*P* value (LCI vs. BLI-b)
All EACs, mean ± SD	12.95 ± 11.86	19.96 ± 7.97	14.47 ± 9.80	0.034^∗^	0.58	0.071
EACs in SSBE, mean ± SD	11.52 ± 7.89	23.20 ± 7.26	13.68 ± 6.76	0.012^∗^	0.67	0.025^∗^
EACs in LSBE, mean ± SD	15.83 ± 18.81	13.48 ± 5.11	16.03 ± 15.52	0.71	0.71	1.00

Wilcoxon signed-rank test, ^∗^*P* < 0.05. WLI: white light imaging; LCI: linked color imaging; BLI-b: blue laser imaging bright; EAC: esophageal adenocarcinoma; SSBE: short-segment Barrett's esophagus; LSBE: long-segment Barrett's esophagus; SD: standard deviation.

## Data Availability

The data used to support the findings of this study are available from the corresponding author upon request.

## Abbreviations

LCI: Linked color imaging
BE: Barrett's esophagus
EAC: Esophageal adenocarcinoma
WLI: White light imaging
BLI-b: Blue laser imaging bright
ΔE∗: Color differences
EGJ: Esophagogastric junction
ESD: Endoscopic submucosal dissection
EMR: Endoscopic mucosal resection
SSBE: Short-segment Barrett's esophagus
LSBE: Long-segment Barrett's esophagus
CLE: Columnar-lined epithelium
IEE: Image-enhanced endoscopy
NBI: Narrow band imaging.
